# Supplementary material for: Health sector readiness for the prevention and control of non-communicable diseases: A multi-method qualitative assessment in Nepal
Source: PLoS One. 2022 Sep 30;17(9):e0272361. doi: 10.1371/journal.pone.0272361 (PMC9524672; doi:10.1371/journal.pone.0272361)
Supplement: Attachment — Submitted filename: Responses to reviewers comments_07.05.docx [file pone.0272361.s002.docx]

July 5, 2022

To

The Academic Editor

PLOS One

*Re: responses to the reviewer’s comments on manuscript entitled "Health sector readiness for the prevention and control of non-communicable diseases: a multi-method qualitative assessment in Nepal (PONE-D-22-10244R1)".*

Dear Sir/Madam,

I would like to express my sincere thanks to you and the editorial team for reviewing the above-mentioned manuscript based on my PhD project. Respecting the feedbacks and comments from academic editor and two reviewers (submitted on 27^th^ May 2022) and editor’s comments (submitted on 5^th^ July, 2022) I have submitted the revised manuscript as explained in the subsequent pages in tabular form.

On behalf of all the authors of this manuscript, please accept our sincere gratitude towards your contribution in this academic endeavor.

Thank you for your cooperation and support

Bhim Prasad Sapkota

PhD Candidate-2019

Center for International Health

Ludwig Maximilian’s University, Munich, Germany

**Point-by-point response to comments made by Academic Editor and two Reviewers (**20 May,2022)

A. **Comments by Academic Editor**

| **SN** | **Comments by academic editor** | **Responses by authors** |
| --- | --- | --- |
| **1.** | Please ensure that your manuscript meets PLOS ONE's style requirements, including those for file naming. The PLOS ONE style templates can be found at  <https://journals.plos.org/plosone/s/file?id=wjVg/PLOSOne_formatting_sample_main_body.pdf> and  <https://journals.plos.org/plosone/s/file?id=ba62/PLOSOne_formatting_sample_title_authors_affiliations.pdf> | The manuscript has been revised as per the PLOS ONE style template. |
| **2.** | Please include a complete copy of PLOS’ questionnaire on inclusivity in global research in your revised manuscript. Our policy for research in this area aims to improve transparency in the reporting of research performed outside of researchers’ own country or community. The policy applies to researchers who have travelled to a different country to conduct research, research with Indigenous populations or their lands, and research on cultural artefacts. The questionnaire can also be requested at the journal’s discretion for any other submissions, even if these conditions are not met.  Please find more information on the policy and a link to download a blank copy of the questionnaire here: <https://journals.plos.org/plosone/s/best-practices-in-research-reporting>. Please upload a completed version of your questionnaire as Supporting Information when you resubmit your manuscript.  We have noted that ethics approval from the ethical committee of the Munich Medical Research School was obtained after data collection was initiated. As PLOS ONE expected all human subject research to have ethics approval prior to data collection, we would be grateful if you could provide some clarification. | 2.1 PLOS’ questionnaire on inclusivity in Global Research has been filled in and will be uploaded as Supporting information (S1 Check list).  As per PLOS One guidance, the following sub-heading has been added under methods and materials:  ‘Inclusivity in global research’  2.2 Ethics approval from the ethical committee of the Munich Medical Research School was obtained on 21.08.2020.  While the policy review (not requiring ethical approval) was initiated in August 2020, data collection (FGDs, IDIs) was only started in October 2020 and completed in December 2020.  Therefore, data collection was initiated after obtaining ethical approval.  This was incorrectly presented in the manuscript as data collection taking place during July-September 2020; this error has been corrected in the revised manuscript. |
| **3.** | In your Data Availability statement, you have not specified where the minimal data set underlying the results described in your manuscript can be found. PLOS defines a study's minimal data set as the underlying data used to reach the conclusions drawn in the manuscript and any additional data required to replicate the reported study findings in their entirety. All PLOS journals require that the minimal data set be made fully available. For more information about our data policy, please see <http://journals.plos.org/plosone/s/data-availability>.  Upon re-submitting your revised manuscript, please upload your study’s minimal underlying data set as either Supporting Information files or to a stable, public repository and include the relevant URLs, DOIs, or accession numbers within your revised cover letter. For a list of acceptable repositories, please see <http://journals.plos.org/plosone/s/data-availability#loc-recommended-repositories>. Any potentially identifying patient information must be fully anonymized.  Important: If there are ethical or legal restrictions to sharing your data publicly, please explain these restrictions in detail. Please see our guidelines for more information on what we consider unacceptable restrictions to publicly sharing data: <http://journals.plos.org/plosone/s/data-availability#loc-unacceptable-data-access-restrictions>. Note that it is not acceptable for the authors to be the sole named individuals responsible for ensuring data access.  We will update your Data Availability statement to reflect the information you provide in your cover letter. | Minimal data sets underlying the (i) policy review, (ii) FGDs and (iii) IDIs are now uploaded as Supporting Information files(S2).  Thank you for updating the information in the data availability statement. |
| **4.** | Please amend either the abstract on the online submission form (via Edit Submission) or the abstract in the manuscript so that they are identical. | The abstract in the manuscript has been shortened to meet the word limit of 300 words. The abstract in the online submission form has been changed accordingly. |

B. **Comments by Reviewer 1**

| **SN** | **Comments by reviewer** | **Author’s responses** |
| --- | --- | --- |
| 1 | The topic is an important one no doubt and the approach to answer the research question is unique. However, the aim of the study needs to be re articulated to be coherent with the methodology used. There seems to be a mismatch- the aim is to assess readiness that is typically assessed by availability of infrastructure, equipment personnel etc. please see WHO tools for service availability and readiness (<https://www.who.int/data/data-collection-tools/service-availability-and-readiness-assessment-(sara)?ua=1>) but the methods are only qualitative. | We have re-articulated the aims of the study as follows:  *…..The aim of this study was to assess the readiness of the Nepalese health sector for the prevention and control of NCDs and their risk factors by exploring the perspectives of policy makers, program managers, and service providers…..* |
| 2 | The choice of a qualitative approach needs some justification- did the authors want to know why there are gaps in implementation or a lack of readiness? The choice of qualitative methods are usually to answer explanatory and exploratory questions - why and how questions..  Since the question is not well articulated the analysis also seems to be inconsistent | The aims of the study (see above) now clearly point to a qualitative study.  We have re-articulated the research question as follows, clearly indicating the three components of the study and the exploratory (how) nature of these components with regards to health sector readiness:  *“the study sought to understand how NCD prevention and control is anchored in Nepalese policy documents, and how different cadres of health workers as well as health policy makers and program managers at provincial and local levels perceive health sector readiness with regards to NCD prevention and control”* |
| 3 | The analysis mentioned is sometimes as thematic, sometimes content analysis and also a framework is mentioned. | We would like to thank the reviewer for this important comment and, in re-reading and making revisions, noticed that our description had not been clear and not 100% accurate.  In short, we did perform content analysis for both the policy review and the analysis of FGDs and IDIs, using the WHO health system building blocks framework as the main categories.  This has been clarified under the heading “Materials and methods” and throughout the manuscript. |
| 4 | Thematic analysis should present in the results the main themes that emerged - headings from the framework can be used but themes need to be presented. What was the main finding is difficult to sense as a reader. | Addressed by our response to comment 3 above. |
| 6 | I would suggest reworking the question to frame a why is the situation the way it is kind of question- and then do a thematic analysis. | Addressed by our response to comment 2 above. |
| 7 | Please remove designations of persons interviewed in table 1 - I assume these would be fairly easy to identify and I find this unethical to mention in the paper. Knowing the designation is not critical to the analysis. | The table has been revised to remove the designation of respondents. |
| 8 | I have missed the codes or coding tree - please include this as an Annexure | codes have been submitted to editor as minimum dataset, named supplementary file (S2) |
| 9 | Only 1 person doing the analysis is a limitation and should be mentioned as such. Another limitation is the lack of other approaches to data- quantitative data on personnel availability, facility assessments. Triangulation of all sources would have added to the study. | Both of these aspects are now being covered in the Discussion, i.e. only one person fully engaging with the analysis (which is now also made transparent under materials and methods) and having used a qualitative approach only. |
| 10 | There are discrepancies in the data- example regarding resource availability- how did the authors deal with the discrepancy- were follow up interviews done to understand the contradictions? | The findings observed in the first interview have been analyzed, discrepancies were described whenever they were observed. No follow up interviews were conducted |
| 11 | Please also state the authors positionality while conducting this research as this would be important to the interpretation of the findings. | A statement about *‘authors positionality’* has been added in the last sub-heading of the methods and materials headings |

C. **Responses to the comments by Reviewer 2**

| **SN** | **Comments by reviewer** | **Author’s responses** |
| --- | --- | --- |
| 1 | Abstract: Needs to be shortened. Second line can be deleted. The building blocks should be in methods and not results. There is too much repetition of the fact that risk factors are not being addressed. The last line can also go. | The abstract has been shortened to keep it less than 300 words. |
| 2 | Introduction can also be made crisper and shorter. In addition, to the section on NCD burden global and Nepal (one para) the next para has to be on global strategies for Prevention and Control of NCDs and their components (rather than listing declarations) and explain the concept of readiness. | The Introduction has been shortened and revised as per the feedback received.  A description of the concept of readiness and health sector readiness has now been added with reference to the literature. |
| 3 | Overall choice of study participants and the methods adopted are appropriate. | Thank you. |
| 4 | Results: The use of health system building block framework is appropriate. Authors may want to revise the sequence in which the result is presented starting with governance & stewardship. Financing and ending with monitoring. Currently one could link the statement of identifiable details of the respondents (place and position) and can be avoided. | The findings were presented in accordance with the order offered through the original WHO health system building blocks publication; each building block has also been renamed (where necessary) in accordance with the original publication. The order, in which the blocks are presented, is consistent across all sections of the manuscript.  The findings have been presented as per the sequence of the building blocks mentioned in the introduction and methods section.  Any details that may contribute to a possible identification of respondents have been removed in the results as well as the tables. |
| 5 | Table 4 which shows the key results need to be organized better (retain the building blocks) to make it more informative. There are many ways that it can be done. For example, they could be split between policy review (can add the number of documents here rather than in text) component to FGD/IDI component. It could also be organized to show the good things and not so good things identified in the review. | Table 4 has been reorganized, with findings now categorized as “findings suggesting health sector readiness” and “findings suggesting a need for improvement in health sector readiness.” |
| 6 | Authors talk about NCD- specific worker and make this as a deficiency. However, more important and better approach is to include NCDs as a basic-package which is being done. This needs to be rethought. | We agree that this represents a more important first step in overcoming the problem of lack of skills in the health workforce. Accordingly, this is now being suggested in the heading ‘discussion’. |
| 7 | Discussion should include some references to other country experiences and also implications for future. | References to the experiences in India, Bangladesh, Uganda, Ghana and Thailand have been added, as per the reviewer’s suggestion.  Two statements about the implication for research and practice have been added in the heading ‘conclusion’. |

D. **Comments by Editor on the Questionnaire explaining Inclusivity in global research (5^th^ July, 2022)**

| **SN** | **Comments/ Issue to be clarified** | **My Response** |
| --- | --- | --- |
| 1. | We have noted that to questions on human subject research, you have answered 'NA' to the questions 'How did members of the local community provide input on the aims of the research investigation, its methodology, and its anticipated outcome(s)? ' | As the study was not targeted to the local community people (that’s why we assume, it is not applicable for this study)  The health workers and health service providers, program managers and policy makers were included in the study.  Before initiation of the FGDs and IDIs study articipants were explained clearly about the objectives,methodology and expected outcomes of the study by using their local language.  (Explained in the page no 2 of S1 checklist ) |
| 2. | 'when engaging with the local community, how did you ensure that the informed consent documents and other materials could be understood by local stakeholders?' | The local community was not engaged in the study.  The study population were health workers, health service providers, health program managers and policy makers.  Informed consent document had been read out in Nepali Language just before the initiation of the FGD s and IDIs to ensure that they understood it clearly.  Moreover, Informed consent form was prepared in Nepali language and given to each study participnats just before starting the FGDs and IDIs.  (Explained in the page no 2 of S1 checklist ) |
